# Effect of Temperature on Biochemical Composition, Growth and Reproduction of the Ornamental Red Cherry Shrimp *Neocaridina heteropoda heteropoda* (Decapoda, Caridea)

**DOI:** 10.1371/journal.pone.0119468

**Published:** 2015-03-13

**Authors:** Carolina Tropea, Liane Stumpf, Laura Susana López Greco

**Affiliations:** 1 Biology of Reproduction and Growth in Crustaceans, Department of Biodiversity and Experimental Biology, FCEyN, University of Buenos Aires, Cdad. Univ. C1428EHA, Buenos Aires, Argentina; 2 IBBEA, CONICET-UBA, Buenos Aires, Argentina; The Evergreen State College, UNITED STATES

## Abstract

The effect of water temperature on biochemical composition, growth and reproduction of the ornamental shrimp, *Neocaridina heteropoda heteropoda*, was investigated to determine the optimum temperature for its culture. The effect of embryo incubation temperature on the subsequent performance of juveniles was also evaluated. Ovigerous females and recently hatched juveniles (JI) were maintained during egg incubation and for a 90-day period, respectively, at three temperatures (24, 28 and 32°C). Incubation period increased with decreasing water temperature, but the number and size of JI were similar among treatments. At day 30 of the 90-day period, body weight and growth increment (GI) at 24°C were lower than those at 28 and 32°C. On subsequent days, GI at 24°C exceeded that at 28 and 32°C, leading to a similar body weight among treatments. These results suggest growth was delayed at 24°C, but only for 30 days after hatching. The lipid concentration tended to be lowest, intermediate and highest at 28, 32 and 24°C, respectively, possibly as a consequence of the metabolic processes involved in growth and ovarian maturation. Protein and glycogen concentrations were similar among treatments. Both the growth trajectory and biochemical composition of shrimps were affected by the temperature experienced during the 90-day growth period independently of the embryo incubation temperature. During the growth period, shrimps reached sexual maturity and mated, with the highest proportion of ovigerous females occurring at 28°C. All the females that matured and mated at 32°C lost their eggs, indicating a potentially stressful effect of high temperature on ovarian maturation. Based on high survival and good growth performance of shrimps at the three temperatures tested over the 90-day period it is concluded that *N. heteropoda heteropoda* is tolerant to a wide range of water temperatures, with 28°C being the optimum temperature for its culture.

## Introduction

Water temperature is one of the most important physical factors affecting survival and growth of decapod crustaceans [1]. Growth rate has been shown to increase with increasing temperature to a maximum, before declining near the upper thermal limit of tolerance [1]. By manipulating this parameter, it may be possible to reduce the time needed to culture economically important species. However, high temperatures also increase mortality, as demonstrated for penaeid shrimps, possibly because less protein is incorporated into body tissues [2,3].

Changes in temperature can affect the metabolic efficiency of an organism [4], which is presumably reflected in its elemental and biochemical composition [5]. In fact, temperature is one of the abiotic factors to which some authors relate seasonal variations in the biochemical composition of decapod crustaceans, such as crayfishes [6–8], marine shrimps [9] and crabs [10]. The effect of temperature on crustacean biochemical composition has also been studied under controlled laboratory conditions of feeding and water quality [11–14]. In particular, an increase in temperature was shown to augment total lipid content in adult males of the penaeid shrimp *Litopenaeus vannamei* [11] and decrease the mean protein content of northern shrimp *Pandalus borealis* larvae [13]. To our knowledge no study has ever been performed to address the possible influence of water temperature on the biochemical composition of freshwater shrimps.

Water temperature also influences survival and development of embryos and larvae of decapods [15,16]. A decrease in the incubation period has been reported as a result of increasing temperature for many species [13,17–21], and for some of them this has been associated with lower survival, higher energy consumption, and even serious deformities of embryos [20,22]. Moreover, the temperature experienced during embryogenesis may influence larval biomass at hatching and subsequent larval development in decapods [23,24]. Therefore, special care must be taken when temperature is manipulated to accelerate embryonic development.

The genus *Neocaridina* comprises freshwater shrimp species native to China, Japan, Korea, Vietnam and Taiwan [25]. Many of these species have been growing in popularity in the aquarium industry over the past years [26, 27]. In particular, *N. heteropoda heteropoda* is popular among freshwater aquarists due to its bright coloration which includes different shades of red (red cherry shrimp), yellow (yellow shrimp), and blue (Neocaridina blue). Despite its potential economic importance as an ornamental shrimp, little quantitative information is available regarding the reproductive and growth performance of this species, and the effects of temperature on these parameters remain unknown.

Based on the considerations mentioned above, the objectives of the present study were (1) to determine the optimum temperature for *N. heteropoda heteropoda* culture by evaluating the effect of this parameter on its reproduction and growth, and (2) to determine if the temperature experienced during embryonic development affects the subsequent growth performance of juveniles. This information will also expand the scarce literature addressing those topics on freshwater crustaceans.

## Materials and Methods

### Experimental rationale

**Fig 1:**
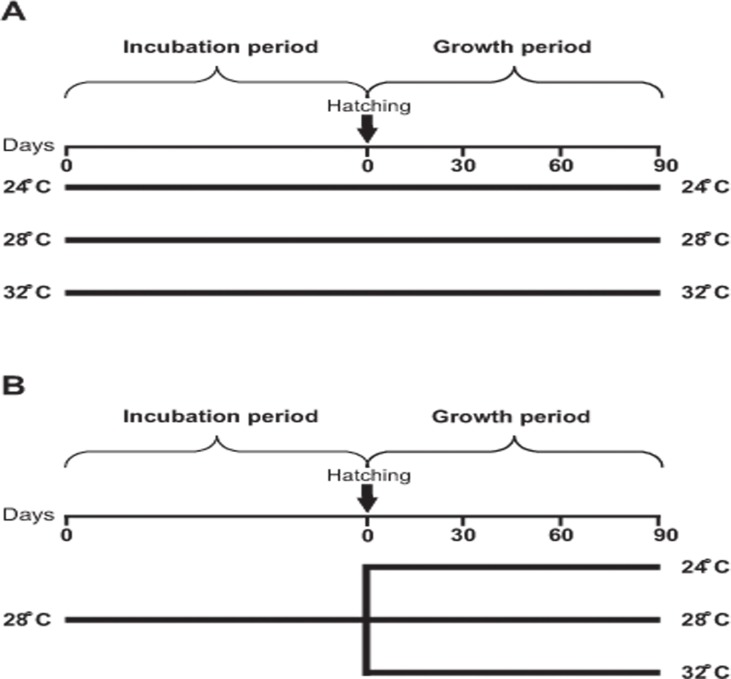
General schedule of the protocols applied in the two experiments performed. In **Experiment 1** (A) *Neocaridina heteropoda heteropoda* juveniles grew at the same temperature at which they were incubated as embryos (24, 28 or 32°C), while in **Experiment 2** (B) embryos were incubated at 28°C, but juveniles grew either at 24, 28 or 32°C. The duration of the *Incubation period* varied according to temperature, while the duration of the *Growth period* was 90 days. Day 0 of the *Incubation period* was the spawning day.

Two experiments were carried out, one of them from April to September, 2013 (**Experiment 1**) and the other one from May to September, 2013 (**Experiment 2**). Fig. 1 shows the general schedule of the treatments and protocols used in both experiments.

Two periods were defined in each experiment: the *Incubation period* and the *Growth period*. During the incubation period of **Exp. 1** temperature was independently set at three different levels (24, 28 and 32°C) in order to evaluate its effect on the duration of the incubation period, actual fecundity, and the size and weight of recently hatched juveniles (objective 1). During the growth period of **Exp. 1** and **Exp. 2** temperature was set at three different levels (24, 28 and 32°C) to evaluate its effect on final weight and size, growth increment, survival, and biochemical composition of shrimps (objective 1). The difference between the two experiments is that in **Exp. 1** juveniles grew at the same temperature at which they were incubated as embryos, while in **Exp. 2** they did not (Fig. 1). Comparing the results of these two experiments allowed us to evaluate the influence of the temperature experienced during embryonic development on the growth performance of juveniles (objective 2).

### Animals

The reproductive stock was obtained on March 2013 from a commercial supplier (Acuamanus Aquarium, Buenos Aires, Argentina) and was placed into plastic aquaria of 33.5 x 25 x 19 cm (35 animals/m^2^) containing 8 L of dechlorinated tap water, under continuous aeration (pH 7.5, hardness 80 mg L^-1^ as CaCO3 equivalents). The temperature was held constant at 27 ± 1°C by water heaters (100 W, precision 1°C), and the photoperiod was 14L:10D. Each aquarium was provided with a small clump (~1.6 g) of Java moss (*Vesicularia* sp.) as shelter. The animals were fed daily *ad libitum* with commercial balanced food for tropical fish (Tetracolor, Tetra GmbH, Germany), with an approximate composition as follows: min. crude protein 47.5%, min. crude fat 6.5%, max. crude fiber 2.0%, max. moisture 6.0%, min. phosphorus 1.5% and min. ascorbic acid 100 mg Kg^-1^.

### Experimental design

**Incubation period**. The reproductive stock was visually inspected twice a day (in the morning and afternoon) to detect the presence of ovigerous females. Once detected they were individually placed in plastic aquaria of 18 x 12.5 x 12 cm containing 1.8 L of dechlorinated tap water, under the same experimental conditions described above (day 0 of the *Incubation period* in Fig. 1). In **Exp. 1** ovigerous females were randomly assigned to aquaria maintained at either 24, 28 or 32 ± 1°C. The two lower temperatures are within the range reported by aquarists for this species, while the higher temperature is near the upper limit of that range (http://www.planetinverts.com/; http://www.theshrimpfarm.com/). In **Exp. 2** all ovigerous females were maintained at a water temperature of 28 ± 1°C (Fig. 1).

All aquaria were cleaned and water was completely replaced once a week; females were visually inspected once a day to determine the hatching date and calculate the duration of the incubation period for each brood. Recently hatched juveniles (juveniles I = JI) were counted to calculate actual fecundity and their mothers were removed from the aquaria and weighed (precision: 0.1 mg).

A sample of 10 to 20 JI was taken from each brood, and the cephalothorax length of each juvenile was measured with the help of a stereomicroscope from the tip of the rostrum to the posterior end of the cephalothorax. A mean value of this variable was calculated for the sample. Then the sample was weighed (wet weight; precision: 0.1 mg) and the individual juvenile weight was calculated by dividing the sample weight by the number of JI. Each brood was a replicate.

**Growth period**. Another sample of 10 JI was taken from each brood to analyze survival and growth during a 90-day period. Each sample was maintained in a plastic aquarium of 18 x 12.5 x 12 cm (444 animals/m^2^), at identical experimental conditions as their mothers had previously been exposed to. In **Exp. 1**, water temperature was the same as during the *Incubation period*, while in **Exp. 2** juveniles were randomly assigned to one of the temperature treatments mentioned above (24, 28 or 32°C; Fig. 1).

Ten broods were evaluated at each water temperature. Juveniles were fed daily *ad libitum* with Tetracolor, and water was completely replaced once a week. It became evident throughout the *Growth period* that shrimps matured and mated (see Results); hence, the presence of ovigerous females was recorded when cleaning the aquaria. We will refer hereinafter to these females as “growth-phase ovigerous females” to distinguish them from the initial broodstock females. Every 30 days, the animals were weighed (precision: 0.1 mg) and mortality was recorded. At the end of the 90-day period shrimps were sacrificed after being cold-anaesthetized at −20°C for 15 minutes and sexed with the help of a stereomicroscope, based on the morphology of the first and second pair of pleopods (Shih and Cai, 2007). Then, final body weight and final cephalothorax length were recorded. A mean value of both variables was calculated for the sample, so that each brood was a replicate. All the animals were stored at -70°C for biochemical analysis.

### Biochemical analysis

Total protein, total lipid and glycogen (expressed in ug/mg) were determined spectrophotometrically in homogenates of all the animals from a replicate, according to the methods described by Bradford (1976) [28], Folch et al. (1957) [29] and Van Handel (1965) [30], respectively. Five replicates of each treatment were randomly selected for this purpose (15 samples for **Exp. 1** and 15 samples for **Exp. 2**), and calculations were performed on a wet weight basis.

For protein determination, samples weighing 100 mg were homogenized in 400 ml of 50 mM Tris-HCl buffer, pH 7.5, and centrifuged at 10,000 x g for 30 minutes in a refrigerated centrifuge (4°C). Total protein was estimated in the supernatant by the Coomassie blue dye method, with bovine serum albumin as standard. Absorbance was read at 595 nm. For lipid determination, samples weighing 200 mg were homogenized in 4 ml of a mixture of methanol and chloroform (2:1, v/v), then mixed with 0.9% NaCl and centrifuged to separate the lipid fraction. Total lipid was quantified by the sulfophosphovanillin method, with extra virgin olive oil (Indalo Clásico; initial concentration: 1 g/ml) diluted with absolute ethanol (1:1000 dilution to give a final concentration of 1 mg/ml) as standard. Absorbance was read at 530 nm. For glycogen determination, samples weighing 100 mg were boiled with 400 μl of KOH 30% for 1 hour. After cooling, glycogen was precipitated with the addition of 75 μl of saturated Na_2_SO_4_ and 1875 μl of absolute ethanol, and centrifugation at 2000 x g for 10 minutes. Then, the precipitate was dissolved in 250 μl of distilled water, and glycogen was measured by the anthrone-reagent method. Rabbit liver (Fluka) was used as standard and absorbance was read at 620 nm.

### Statistical Analyses

The following variables were calculated to evaluate the reproductive and growth performance of shrimps:

- Actual fecundity: the total number of hatched juveniles/female. This variable was calculated only for the *Incubation period* of **Exp. 1**.
- Percentage of growth-phase ovigerous females: 100*(number of ovigerous females/total number of females). This variable was calculated for the *Growth period* of **Exp. 1** and **2**.
- Growth Increment (GI): 100*((BW_f_−BW_i_)/ BW_i_), where BW_f_ and BW_i_ are the final and initial body weights respectively, for the periods 0–30, 30–60 and 60–90 days within the *Growth period*. This variable was calculated for **Exp. 1** and **2**.

Broods smaller than 20 were not used to analyze growth performance, but they were considered to evaluate all the other variables mentioned.

**Fig 2:**
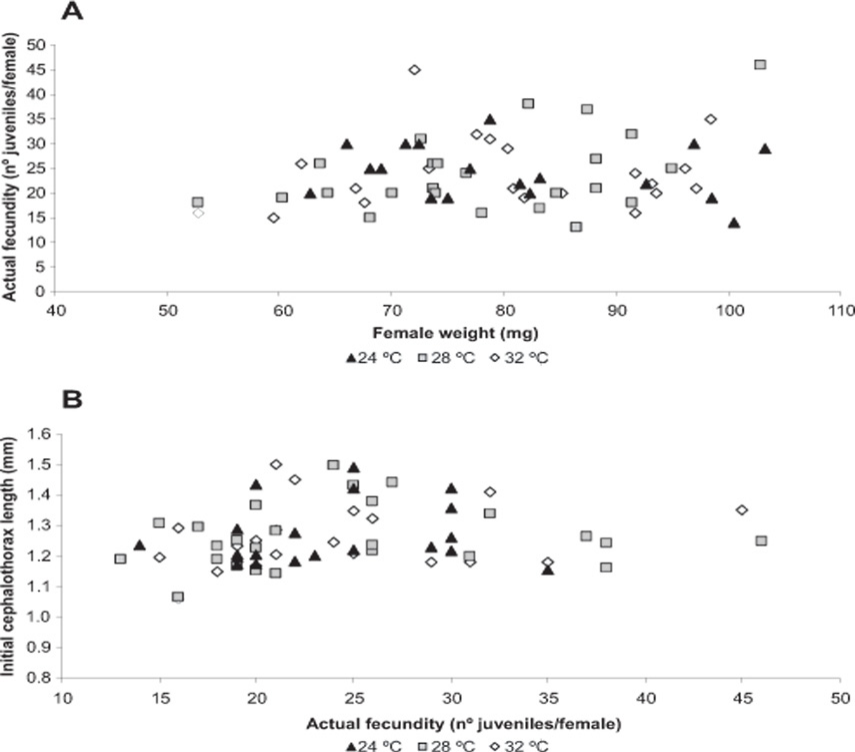
Relationship between actual fecundity and female weight (A) and between juvenile initial cephalothorax length and actual fecundity (B). *Neocaridina heteropoda heteropoda* females were kept at three different water temperatures (24, 28 or 32°C) over the incubation period, from the spawning day to the hatching day. The number of females *per* treatment was 19 at 24°C, 26 at 28°C and 20 at 32°C. No statistical relationships were found between the variables analyzed (*P* > 0.05).

The variables recorded in the *Incubation period* of **Exp. 1** (weight and survival of ovigerous females, duration of the incubation period, actual fecundity, cephalothorax length and body weight of JI) were compared among temperature treatments (24, 28 and 32°C) using a one-way analysis of variance (ANOVA). Some variables recorded in the *Growth period* of **Exp. 1** and **Exp. 2** (initial and final cephalothorax lengths, survival, and biochemical composition of shrimps, and the proportion of females/males) were also compared among treatments using one-way ANOVA. To account for multiple testing, we used the Bonferroni correction and considered significant only those comparisons for which P < 0.05/11 = 0.005, with 11 being the total number of ANOVAs performed. This correction is known to be conservative and thus “over-corrected” the raw P values. In addition, linear regression was used to analyze actual fecundity *versus* maternal weight and cephalothorax length of JI *versus* actual fecundity. Repeated measures one-way ANOVA was performed to test for differences in body weight and GI among treatments, followed by the multiple comparison LSD Fisher test when significant differences were found. The Fisher exact test was used to compare the percentage of growth-phase ovigerous females among treatments. The replicate unit was each ovigerous female (for the female weight and survival in the *Incubation period*), and each brood (for the cephalothorax length and weight of JI in the *Incubation period*, the initial and final cephalothorax lengths, initial and final weights, GI, survival, and biochemical composition of shrimps, and the proportion of females/males in the *Growth period*). The number of replicates *per* treatment for each variable is given in Table 1 and Fig. 2. The results *per* treatment are presented as mean ± SE. All tests were carried out with STATISTICA version 8.0 [31].

**Table 1:** Effect of temperature (24, 28 and 32°C) on reproduction and growth of the freshwater shrimp *Neocaridina heteropoda heteropoda* during the *Incubation periods* and *Growth periods* of Experiments 1 and 2.^1^ ^,^ ^2^ ^,^ ^3^

Variable^2^	Experiment 1				Experiment 2			
	24°C	28°C	32°C	*P*-value	24°C	28°C	32°C	*P*-value
***Incubation period***								
Duration, days	20.84±0.26^a^(19)	14.62±0.19^b^(26)	12.40±0.28^c^(20)	<0.001	-	-	-	-
AF, n° juveniles hatched/female	24.16±1.22^a^(19)	24.35±1.62^a^(26)	24.05±1.65^a^(20)	0.990	-	-	-	-
IBW, mg	0.20±0.01^a^(19)	0.21±0.01^a^(26)	0.20±0.01^a^(20)	0.317	-	-	-	-
ICL, mm	1.27±0.02^a^(19)	1.26±0.02^a^(26)	1.26±0.02^a^(20)	0.863	-	-	-	-
***Growth period***								
ICL, mm	1.31±0.03^a^(9)	1.30±0.03^a^(10)	1.25±0.03^a^(9)	0.327	1.27±0.01^a^(10)	1.26±0.01^a^(10)	1.30±0.01^a^(9)	0.550
FCL, mm	6.27±0.14^a^(9)	6.70±0.16^a^(10)	6.65±0.11^a^(9)	0.076	6.28±0.17^a^(10)	6.85±0.04^a^(10)	6.95±0.13^a^(9)	0.003
Survival, %	82.22±4.34^a^(9)	83.00±6.16^a^(10)	63.33±4.74^a^(9)	0.024	65.00±4.53^a^(10)	85.00±3.42^a^(10)	72.22±7.38^a^(9)	0.039
Sexual ratio, % female:male	60.2:39.8^a^(9)	54.2:45.8^a^(10)	51.6:48.3^a^(9)	0.586	54.1:45.9^a^(10)	60.8:39.2^a^(10)	56.9:43.1^a^(9)	0.762
OF, %	25.00^a^	100.00^b^	14.00^a^	*P* ^3^	26.5^a^	72.7^b^	25.0^a^	*P* ^3^
Lipid concentration, ug/mg	5.01±0.40^a^(5)	3.71±0.56^a^(5)	4.84±1.00^a^(5)	0.335	5.63±0.44^a^(5)	3.09±0.28^a^(5)	4.11±0.64^a^(5)	0.009
Protein concentration, ug/mg	26.82±1.33^a^(5)	25.12±2.31^a^(5)	29.38±1.38^a^(5)	0.269	29.03±2.55^a^(5)	24.99±3.68^a^(5)	31.14±2.43^a^(5)	0.356
Glycogen concentration, ug/mg	0.46±0.07^a^(5)	0.48±0.07^a^(5)	0.53±0.05^a^(5)	0.812	0.36±0.06^a^(5)	0.35±0.08^a^(5)	0.48±0.10^a^(5)	0.440

^1^Comparisons were made among temperature treatments within each experiment. The number between brackets represents the number of replicates used to calculate each variable. Within a row means without a common superscript differ (*P* < 0.05).

^2^AF = actual fecundity; IBW = initial body weight; ICL = initial cephalothorax length; FCL = final cephalothorax length; OF = percentage of ovigerous females.

^3^ *P*-values from the Fisher exact test: *P* = 0.000 for the comparison of OF between 24°C and 28°C; *P* = 0.118 for the comparison of OF between 24°C and 32°C; *P* = 0.000 for the comparison of OF between 28°C and 32°C.

## Results

### Incubation period

In **Exp. 1**, the weight (79.3 ± 1.6 mg) and survival (95.4%) of ovigerous females at the end of the *Incubation period* were similar (*P* > 0.05) among treatments. The mean duration of the incubation period increased significantly (*P* < 0.005) with decreasing water temperature, from 12 days at 32°C to almost the double (21 days) at 24°C, while actual fecundity was similar (*P* > 0.005) among treatments, and reached a mean (±SE) value of 24.2 (±0.1) juveniles that hatched *per* female (Table 1). No statistical relationship (*P* > 0.05) was found between actual fecundity and female weight, and between the cephalothorax length of JI and actual fecundity (Fig. 2). In addition, the cephalothorax length and body weight of JI did not vary (*P* > 0.005) with the embryo incubation temperature, with mean values being 1.26 ± 0.02 mm and 0.20 ± 0.01 mg, respectively (Table 1).

In **Exp. 2**, the weight of the ovigerous females (58.4 ± 1.9 mg) and the cephalothorax length (1.29 ± 0.03 mm) and body weight (0.15 ± 0.01 mg) of JI were similar (*P* > 0.005) among treatments (Table 1).

### Growth period

**Fig 3:**
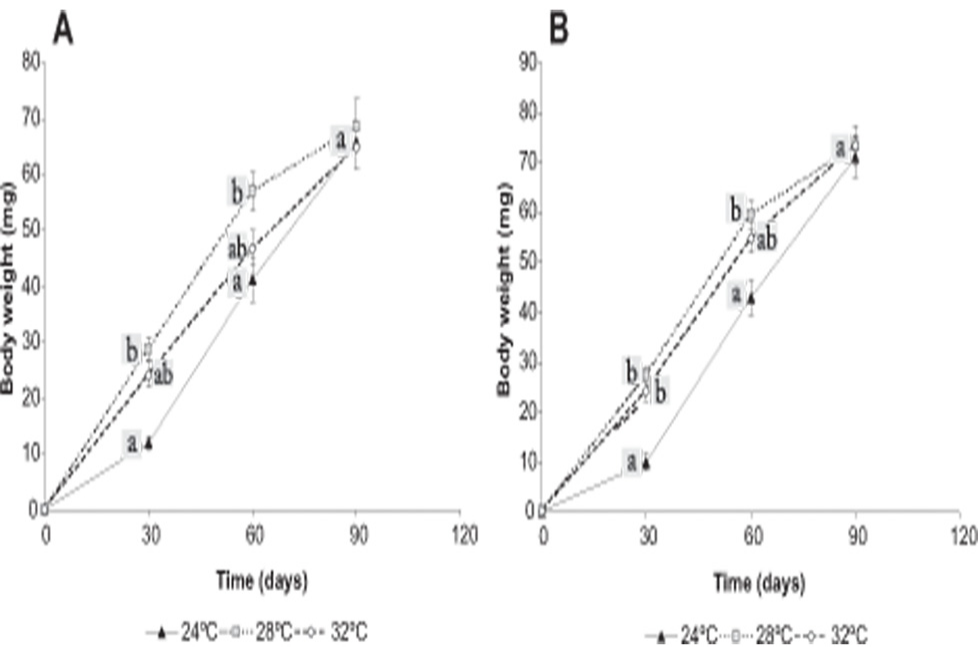
Body weight (mean±SE) of shrimps during the *Growth period* of Experiment 1 (A) and Experiment 2 (B). *Neocaridina heteropoda heteropoda* juveniles were reared at three different water temperatures (24, 28 or 32°C) over a 90-day period, and the body weight was recorded every 30 days. Different letters at days 30 and 60 indicate statistically significant differences among treatments (*P* < 0.05); the letter “a” at day 90 indicates the absence of statistically significant differences among treatments (*P* > 0.05). In both experiments, body weight of shrimps maintained at 24°C was lower than that of shrimps maintained at 28°C at days 30 and 60, but this difference was no longer significant at day 90.

**Fig 4:**
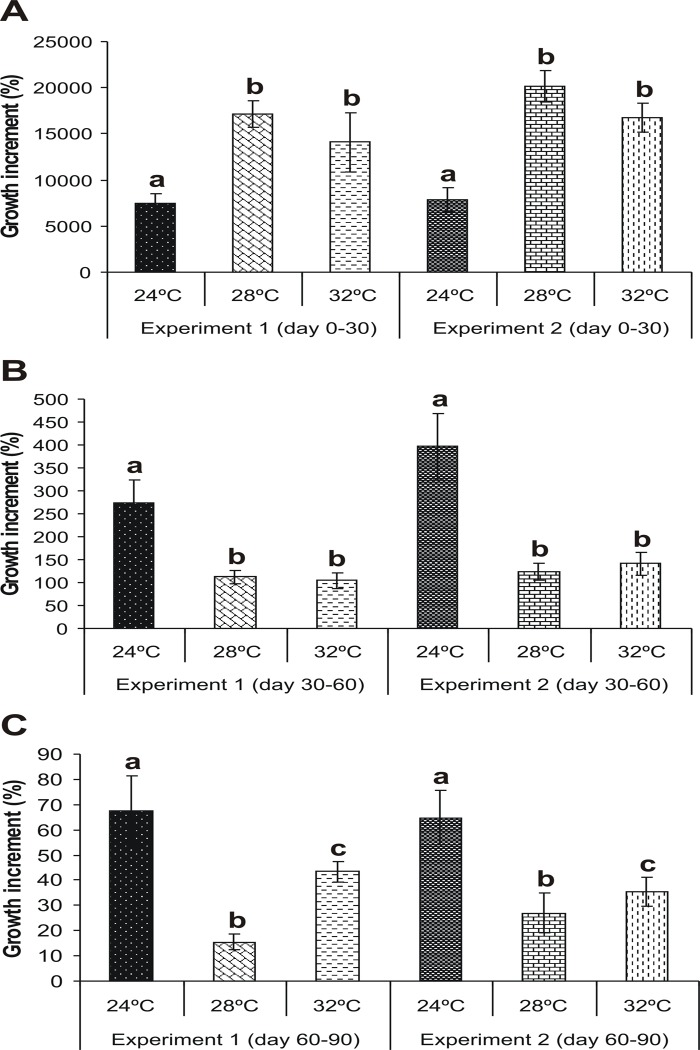
Growth increment (mean±SE) of shrimps during the *Growth period* of Experiment 1 and Experiment 2. *Neocaridina heteropoda heteropoda* juveniles were reared at three different water temperatures (24, 28 or 32°C) over a 90-day period and the growth increment was calculated from day 0 to day 30 (A), from day 30 to day 60 (B), and from day 60 to day 90 (C). Different letters indicate statistically significant differences among treatments for each experiment (*P* < 0.05). In both experiments, the growth increment of shrimps maintained at 24°C was lower than that of shrimps maintained at 28 and 32°C at day 30, but this was reversed on subsequent days.

At days 30 and 60 of the *Growth period* of both experiments body weight was lowest, highest and intermediate (*P* < 0.05) in juveniles maintained at 24, 28 and 32°C, respectively. However, at day 90 this variable was similar (*P* > 0.05) among treatments (Fig. 3). The growth increment (GI) was significantly lower (*P* < 0.05) at 24°C than at the other temperatures tested over the first 30 days. This was completely reversed during the following 60 days, with the GI of shrimps at 24°C exceeding that of shrimps at 28 and 32°C (*P* < 0.05). At the end of the *Growth period*, GI was lowest, intermediate and highest (*P* < 0.05) at 28, 32 and 24°C, respectively (Fig. 4).

In both experiments, the final cephalothorax length of shrimps maintained at 24°C was lower than that of shrimps maintained at 28 and 32°C, but this difference was not statistically significant (*P* > 0.005; Table 1). A tendency was observed towards highest survival at 28°C in both experiments and lowest survival at 32°C in **Exp. 1** and at 24°C in **Exp. 2** (*P* > 0.005; Table 1).

**Fig 5:**
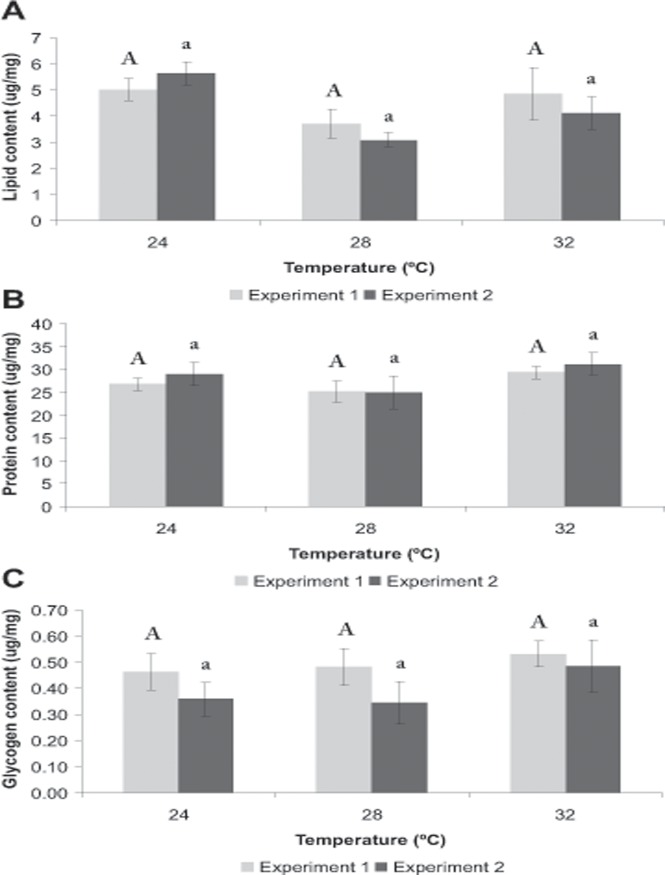
Biochemical composition of shrimps during the *Growth period* of Experiment 1 and Experiment 2. *Neocaridina heteropoda heteropoda* juveniles were reared at three different water temperatures (24, 28 or 32°C) over a 90-day period and the lipid (A), protein (B), and glycogen (C) concentrations (mean±SE) were measured at the end of that period. Different letters indicate statistically significant differences among treatments for each experiment (*P* < 0.05). In both experiments, the protein and glycogen concentrations were similar among treatments, while the lipid concentration tended to be lowest in shrimps maintained at 28°C and highest in shrimps maintained at 24°C.

The lipid concentration was highest at 24°C, intermediate at 32°C and lowest at 28°C for both experiments, but this difference was not statistically significant (*P* > 0.005). The protein and glycogen concentrations were similar (*P* > 0.005) among treatments for both experiments. The former reached mean values of 27.1 and 28.4 ug/mg for **Exp. 1** and **2**, respectively, while the latter reached mean values of 0.5 and 0.4 ug/mg for **Exp. 1** and **2**, respectively (Table 1; Fig. 5).

Females were identified by their developed yellow/green ovaries, which were visible through the carapace from an approximate weight of 40 mg. Growth-phase ovigerous females were observed at the three temperatures tested from day 50 (weight range: 60 to 120 mg), with the proportion being highest (*P* < 0.05) at 28°C (Table 1). In view of the fact that all the growth-phase ovigerous females at 32°C lost their eggs, second-generation juveniles were obtained only at 28 and 24°C. In both experiments, the proportions of females/males at the end of the *Growth period* were similar (*P* > 0.005) among treatments, but females tended to be in a higher proportion than males (Table 1).

## Discussion

Temperature had a clear influence on the duration of the incubation period for *N. heteropoda heteropoda*, with the developmental time of embryos (*Incubation period* of **Exp. 1**) being accelerated by increasing temperature. This is in agreement with previous findings for other decapod crustaceans [15,19–21]. Some studies have reported negative relationships between incubation temperature and size/biomass of larvae at hatching, and between temperature and conversion efficiency of yolk reserves in developing embryos of marine decapods [19,32,33]. In contrast, the size and weight of recently hatched juveniles of the freshwater shrimp in the present study were similar and independent of the embryo incubation temperature. This suggests that temperature had no effect on either the rate of yolk consumption or on the efficiency in which yolk was converted into tissue. Even though egg survival was not measured, we can infer that it was not affected by temperature, given that (1) eggs were laid by all females (from the reproductive stock) at the same water temperature (27 ± 1°C), (2) females were then randomly assigned to each treatment, and (3) the number of juveniles that hatched *per* female (actual fecundity) was similar among temperatures.

In addition, present results showed that actual fecundity was independent of female weight over a range of 50–120 mg under three different temperatures. This coincides with previous findings in the marine shrimp *P*. *borealis* [13], but disagrees with studies on other freshwater crustaceans, including two shrimp species [26,34,35].

On the other hand, body weight was similar among treatments at the end of the *Growth period* in both experiments. This seems to disagree with many studies on other commercially important freshwater decapods that reported an increased growth with increasing temperature within the corresponding optimal temperature range [1]. However, Wang et al. (2008) [36] found that temperature had no effect on growth of the oriental river prawn *Macrobrachium nipponense* juveniles over a range of 22–32°C, and that growth declined only at lower temperatures (16 and 20°C). On this basis, it seems that *N. heteropoda heteropoda* is highly tolerant to a wide range of water temperatures, with growth being possibly affected at more extreme temperatures than the ones evaluated in the present study. Still, it should be noted that even though the weight and size of juveniles were similar at hatching, their growth trajectories throughout the *Growth period* did differ among treatments. The GI, and consequently body weight, of juveniles maintained at 24°C was lower during the first 30 days than that of juveniles maintained at 28 and 32°C, independently of the embryo incubation temperature. However, during the following 60 days of the 90-day period, a gradual recovery was observed in shrimp growth at 24°C, which led to a final body weight similar to that of shrimps from the other treatments. These results suggest that 24°C delays the growth of *N. heteropoda heteropoda* juveniles for the first 30 days after hatching. Other authors have also reported delayed larval development at lower temperatures for the marine shrimps *Penaeus semiculcatus* [22] and *P*. *borealis* [37].

Since the growth performance of shrimps was similarly affected by temperature in both experiments, it seems that the conditions experienced by embryos prior to hatching (incubation temperatures: 24, 28 and 32°C in **Exp. 1**, and 28°C in **Exp. 2**) had no influence on growth, at least for the evaluated temperature range.

Interestingly, the GI of shrimps at 28°C was found to decrease from day 30 onward. It is well known that ovarian maturation and somatic growth are antagonistic processes from an energetic point of view. The energy costs of ovarian maturation are high due to the increase in biosynthetic work, which supports the lecithotrophic strategy of the embryos [38]. On this basis, a possible explanation for the decrease in GI may be that females maintained at 28°C allocated a greater amount of energy towards reproduction than towards growth compared to females at 24 and 32°C, as evidenced by the highest proportion of growth-phase ovigerous females being obtained at 28°C.

At 32°C, ovaries matured and females laid eggs during the *Growth period*, but the eggs were lost in all cases, even though they were fertilized. However, when the initial broodstock females laid eggs at 27 ± 1°C and were then transferred to 32°C (*Incubation period* of **Exp. 1**), the eggs were not lost. This suggests a potential stressful effect of the high temperature on ovarian maturation (i.e., the transfer of biochemical reserves to oocytes) rather than on embryonic development or egg attachment to the pleopods. In this sense, Fischer et al. (2009) [39] found that the energetic investment per egg (measured as dry mass, carbon and nitrogen content, and volume) was negatively correlated to the temperature experienced by the female crab *Cancer setosus* in the time prior to egg-laying.

With respect to the biochemical composition of *N. heteropoda heteropoda* individuals, temperature was found to have no effect on the concentrations of glycogen and protein at the end of the *Growth periods* of both experiments. This finding may be explained by the fact that the type of diet, food availability and water quality, which are some of the factors influencing the storage-mobilization cycle of glycogen and the protein concentration in crustaceans [40–42], were similar among treatments throughout that period. At the three temperatures evaluated, protein was the most abundant biochemical component, while glycogen represented a minor fraction of body composition, in agreement with results previously reported for the eggs and early developmental stages of the freshwater shrimp *M*. *americanum* [21], and the estuarine prawn *Farfantepenaeus paulensis* [43]. With respect to lipids, a tendency towards higher levels was found in shrimps grown at 24°C in both experiments. This is in line with previous results that reported decreasing consumption rates of lipids with decreasing incubation temperatures in embryos of the freshwater crayfish *Cherax quadricarinatus* [20] and the prawn *M*. *americanum* [21]. In the present study, the lower concentration of lipids in shrimps reared at 28 and 32°C with respect to that of shrimps at 24°C, may be a consequence of an increase in all the metabolic processes involved in growth [20,22,44]. In this sense, Childress et al. (1990) [45] showed for several species of benthic deep-sea decapod crustaceans that metabolic rates decline with decreasing temperature. Higher metabolic rates at higher temperatures are accompanied by an increase in energy demand, which is mainly supplied by lipids in aquatic organisms [46]. The lowest lipid concentration in shrimps reared at 28°C may also be explained by the fact that reproduction was more pronounced at that temperature than at the other temperatures tested. In this sense, several studies have demonstrated that during periods of high energy demand, such as oogenesis, there is a pronounced degradation of lipids for yolk synthesis, which is the major source of nutrients for the developing embryo [47,48]. As for body weight and GI, biochemical composition patterns were similar in both experiments, further indicating that the temperature experienced during embryonic development did not affect the subsequent growth performance of *N. heteropoda heteropoda*.

During the 90-day *Growth period*, the life cycle of this species was completed as evidenced by the fact that juveniles reached sexual maturity and mated. For this reason we consider that survival was good in all treatments, with maximum values obtained at 28°C in both experiments. This parameter tended to be lowest at 24°C when the embryo incubation temperature was 28°C (**Exp. 2**), further indicating a possible stressful effect of low temperature on shrimp performance.

### Conclusions

Present results show that *N. heteropoda heteropoda* has high tolerance to a wide range of water temperatures (24–32°C), as evidenced by its high survival and good growth performance over a 90-day period. The tolerance of this species to that temperature range was lower in terms of its reproductive performance. The conditions experienced by embryos seem to have no effect on the patterns of growth and biochemical composition of shrimps at different temperatures. Taking into account all these variables it is concluded that the optimum temperature for juveniles to grow, mature and breed is around 28°C. However, this may lead to the physiological exhaustion of shrimps, as evidenced by the lowest lipid concentration found at that temperature at the end of the *Growth period*. Once females lay eggs they can be exposed to higher temperatures, in order to accelerate embryonic development without affecting juvenile size at hatching. Further studies, evaluating the biochemical composition of eggs laid by females maturing at different water temperatures are necessary to confirm the potential negative effect of temperature on maternal egg provision. In addition, the analysis of egg biochemical composition throughout embryonic development may confirm an absence of temperature effect on the rate of yolk consumption for this and other freshwater crustaceans.
